# DeepSeek vs. ChatGPT: prospects and challenges

**DOI:** 10.3389/frai.2025.1576992

**Published:** 2025-06-19

**Authors:** Inhye Jin, Jonathan A. Tangsrivimol, Erfan Darzi, Hafeez Ul Hassan Virk, Zhen Wang, Jan Egger, Sean Hacking, Benjamin S. Glicksberg, Markus Strauss, Chayakrit Krittanawong

**Affiliations:** ^1^Yeungnam University College of Medicine, Daegu, Republic of Korea; ^2^Department of Neurosurgery, The Loyal and Edith Davis Neurosurgical Research Laboratory, Barrow Neurological Institute, St. Joseph’s Hospital and Medical Center, Phoenix, AZ, United States; ^3^Department of Neurosurgery, Chulabhorn Hospital, Chulabhorn Royal Academy, Bangkok, Thailand; ^4^MIT Computer Science and Artificial Intelligence Laboratory, Massachusetts Institute of Technology, Cambridge, MA, United States; ^5^Harrington Heart and Vascular Institute, Case Western Reserve University, University Hospitals Cleveland Medical Center, Cleveland, OH, United States; ^6^Robert D. and Patricia E. Kern Center for the Science of Health Care Delivery, Mayo Clinic, Rochester, MN, United States; ^7^Institute for Artificial Intelligence in Medicine, University Hospital Essen (AöR), Essen, Germany; ^8^Department of Pathology, NYU Grossman School of Medicine, New York, NY, United States; ^9^Hasso Plattner Institute for Digital Health, Icahn School of Medicine at Mount Sinai, New York, NY, United States; ^10^Department of Cardiology I-Coronary and Periphal Vascular Disease, Heart Failure Medicine, University Hospital Muenster, Muenster, Germany; ^11^Department of Cardiology, Sector Preventive Medicine, Health Promotion, Faculty of Health, School of Medicine, University Witten/Herdecke, Hagen, Germany; ^12^HumanX, Delaware, DE, United States

**Keywords:** artificial intelligence, DeepSeek-R1, reinforcement learning, open-source, ChatGPT

## Abstract

DeepSeek has introduced its recent model DeepSeek-R1, showing divergence from OpenAI’s ChatGPT, suggesting an open-source alternative to users. This paper analyzes the architecture of DeepSeek-R1, mainly adopting rule-based reinforcement learning (RL) without preliminary supervised fine-tuning (SFT), which has shown better efficiency. By integrating multi-stage training along with cold-start data usage before RL, the model can achieve meaningful performance in reasoning tasks along with reward modeling optimizing training process. DeepSeek shows its strength in technical, reasoning tasks, able to show its decision-making process through open source whereas ChatGPT shows its strength on general tasks and areas requiring creativeness. Despite the groundbreaking developments of both models, there is room for improvement in AI landscape and matters to be handled such as quality of data, black box problems, privacy management, and job displacement. This paper suggests the future of AI, expecting better performance in multi-modal tasks, enhancing its effectiveness in handling larger data sets, enabling users with improved AI landscapes and utility.

## 1 Introduction

Large Language Models (LLMs) have evolved rapidly, enabling a variety of real-world applications in fields ranging from healthcare to software development (Bi et al., 2024; Guo et al., 2024; Vaswani et al., 2017). Among these, ChatGPT—a proprietary and widely adopted LLM—has showcased strengths in general-purpose conversation, content generation, and user-friendliness (Anthropic, 2024; Google, 2024; OpenAI, 2022, 2023, 2024; Wang et al., 2024). By contrast, DeepSeek-R1 is an emerging open-source alternative developed with a cost-focused, reasoning-oriented architecture (Guo et al., 2025; Shao et al., 2024; Wang et al., 2023).

This paper compares DeepSeek-R1 and ChatGPT precisely because they exemplify two increasingly important development philosophies: (1) an open-source approach focused on specialized reasoning tasks and transparent decision-making (DeepSeek-R1), and (2) a closed-source, commercially maintained system built for broad language coverage and polished, general-purpose interactions (ChatGPT). By highlighting how these models differ in training strategy, cost structure, licensing, and core capabilities, we show why an open-source, reinforcement-learning-centric framework (DeepSeek-R1) may prove advantageous in tasks such as high-stakes technical problem-solving or budget-sensitive enterprise deployments, whereas ChatGPT may still be more appropriate for consumer-facing applications that prioritize fluency, adaptability, and ease of use.

Readers can thus draw two main lessons: First, the choice of model depends as much on organizational requirements (e.g., transparency needs, computing budget) as on raw performance metrics. Second, aligning a model’s technical architecture with its intended usage domain (e.g., code generation vs. customer support) can yield significant gains in efficiency and effectiveness. By focusing on these design and licensing contrasts, the comparison here aims to clarify how academic researchers, industry practitioners, and policy makers might navigate the ever-growing ecosystem of LLMs.

The objective of this study is to compare multiple aspects of the two models by first examining the core architecture of each model, strategies of reinforcement learning and its training process. After going through basic structure, next section presents comparative evaluation such as benchmark comparisons, strengths and limitations, which highlights their respective competitiveness. This paper concludes by suggesting improvements that could lead to further advancements in AI models in both open-source and closed-source LLMs.

The remainder of this paper is structured as follows: Section 2 provides a detailed examination of the architectural frameworks of both DeepSeek-R1 and ChatGPT, including their transformer foundations and specialized components. Section 3 explores the training methodologies employed by both models, with particular focus on DeepSeek’s reinforcement learning approach and ChatGPT’s human feedback mechanisms. Section 4 presents a comprehensive comparison of the models across various dimensions including performance metrics, transparency, and ethical considerations. Section 5 analyzes the specific strengths and limitations of DeepSeek relative to ChatGPT, while Section 6 addresses broader challenges facing both models within the current AI landscape. Finally, Section 7 concludes with insights on future directions and potential implications for researchers, practitioners, and policymakers navigating the evolving ecosystem of large language models.

### 1.1 Model architectures, DeepSeek, and ChatGPT

#### 1.1.1 Overview

Both DeepSeek and ChatGPT are built upon transformer architecture, which processes input text in parallel by utilizing self-attention mechanisms.

#### 1.1.2 DeepSeek

DeepSeek-R1 is built upon a transformer framework that incorporates techniques like Mixture of Experts and Multi-Head Latent Attention. These additions help distribute computational effort more efficiently by activating only a fraction of the model’s total parameters during inference, leading to lower memory usage and cost. The training process begins with a small-scale cold-start fine-tuning step that makes use of high-quality data, after which the model undergoes reasoning-oriented reinforcement learning. This multi-stage approach guides DeepSeek toward solutions that are both accurate and clearly formatted, thanks to rules that reward mathematical correctness, coding precision, and the inclusion of structured reasoning tags. Because the entire system operates under an open-source license, developers can inspect and modify the underlying code for customization, although this openness may place some burden on users who need to manage model updates or adapt the framework to different computing environments.

#### 1.1.3 ChatGPT

ChatGPT is built upon a transformer backbone in which all parameters remain active for any given token (a dense architecture). Like DeepSeek-R1, ChatGPT is trained in multiple stages, beginning with large-scale unsupervised pre-training on diverse text corpora. This phase yields broad language understanding and generative capabilities.

After pre-training, OpenAI applies *supervised fine-tuning (SFT)*, where ChatGPT learns more structured responses by examining labeled examples of human–machine interactions. The model is further refined through *Reinforcement Learning from Human Feedback (RLHF)*, in which human annotators rank outputs based on accuracy, helpfulness, and appropriateness. ChatGPT thus iteratively aligns its responses with real-world user preferences.

#### 1.1.4 Parameter and architectural details

While OpenAI has not publicly disclosed the exact number of parameters, ChatGPT is known to have billions of trainable weights in a fully dense layout, meaning the entire parameter set is engaged during token prediction. This provides strong general-purpose performance and can generate fluent, context-aware outputs; however, it typically consumes more computing resources and memory than a mixture-of-experts approach.

#### 1.1.5 Strengths and limitations

Thanks to the broad pre-training corpus and extensive RLHF, ChatGPT can tackle creative language tasks (e.g., storytelling, brainstorming) and demonstrate robust conversational coherence. However, its closed-source nature means developers have limited visibility into its underlying code or decision-making steps. Additionally, ChatGPT’s generalist design can lead to “hallucinations” (Emsley, 2023; Hamid, 2024) in highly specialized or deeply technical domains if not carefully guided by user prompts.

#### 1.1.6 Comparison with DeepSeek-R1

While ChatGPT excels in user-friendly dialogue and a wide range of language tasks, it may carry a higher computational footprint and less modular transparency compared to DeepSeek. In enterprise settings needing cost-conscious, reasoning-intensive, or openly customizable solutions, DeepSeek may be more suitable. Conversely, ChatGPT can be advantageous for consumer-facing applications where fluent conversation, broad language coverage, and minimal tuning are critical. This comparative context, alongside the DeepSeek architecture described above, demonstrates how design trade-offs emerge between dense versus mixture-of-experts approaches, as well as open-source versus closed-source licensing models.

Both models strive to address a range of use cases, yet they differ in fundamental priorities. DeepSeek focuses on cost-effectiveness, transparent design, and specialized reasoning tasks, whereas ChatGPT’s development pathway emphasizes broad coverage, polished natural-language generation, and user-friendly conversation. By discussing these distinctions side by side, subsequent sections can more effectively compare how each system’s design and training methods shape performance, cost structure, and real-world applicability.

### 1.2 DeepSeek architecture

#### 1.2.1 Overview

DeepSeek-R1 is built on a transformer-centric foundation (Vaswani et al., 2017; Liu et al., 2024; Shao et al., 2024) enriched by two key innovations—Mixture of Experts (MoE) and Multi-Head Latent Attention (MLA). The underlying motivation is to address common bottlenecks in large language models, namely high computational requirements and memory overhead. By partitioning parameters and attention mechanisms more intelligently, DeepSeek-R1 aims to sustain strong performance for complex tasks without making the model prohibitively expensive to train or deploy. This approach emerges from growing recognition that simply scaling parameter counts yields diminishing returns unless accompanied by equally sophisticated strategies for parameter management and attention efficiency.

Specifically, MoE restricts the number of parameters actively used for each input token, whereas MLA focuses on compressing and streamlining the attention-related data structures (e.g., key–value caches) across multiple attention heads (Shao et al., 2024; Shazeer, 2019; Ainslie et al., 2023). DeepSeek’s architecture thus embodies a strategic compromise: it maximizes representational capacity for harder tasks while keeping the overall compute load within practical limits. As such, the model can tackle extensive sequences or advanced reasoning tasks in a manner that is more cost-effective than many purely dense transformer architectures.

#### 1.2.2 Transformer model

At its core, DeepSeek-R1 still follows the conventional transformer pipeline, which processes input sequences in parallel rather than strictly sequentially (Vaswani et al., 2017; Liu et al., 2024; Shao et al., 2024; Hamid, 2023). By leveraging self-attention, transformers capture contextual dependencies across entire text passages, making them particularly adept at tasks such as language modeling, translation, or summarization. In DeepSeek, the canonical transformer building blocks—namely the feed-forward network (FFN) and multi-head attention (MHA)—remain central. However, they are extended by specialized submodules to address issues like redundant parameter usage and inefficient scaling.

In practice, the feed-forward network handles local transformations on each token, while the attention module learns global relationships among tokens. The multi-head design partitions attention into multiple subspaces, which helps the model focus on different semantic or syntactic features simultaneously. For DeepSeek-R1, integrating MoE and MLA on top of this baseline allows the model to selectively activate only the most relevant parameters for each token (MoE) while also minimizing memory strain through more efficient caching (MLA). This layered strategy transforms the traditional transformer into a more adaptive, cost-conscious system, aligning with DeepSeek’s overarching emphasis on specialized reasoning and modular extensibility.

#### 1.2.3 Mixture of Experts (MoE)

One of DeepSeek-R1’s core architectural innovations lies in its Mixture of Experts module (Dai et al., 2024; Liu et al., 2024; Lepikhin et al., 2020), which effectively distributes the model’s parameters across multiple specialized subnetworks called “experts.” Instead of relying on a single, monolithic parameter space, the model deploys these experts selectively: a gating mechanism assigns each incoming token to the expert or group of experts best suited to handle it. In doing so, the model prevents all parameters from being active at every inference step, thereby reducing redundant computations. DeepSeek’s implementation reportedly taps into only about 5.5% (37 billion) of its total 671 billion parameters per token (Liu et al., 2024), illustrating how significantly MoE can slash the active compute footprint.

Beyond mere efficiency gains, MoE potentially boosts model accuracy by allowing certain experts to evolve finely tuned knowledge for specific domains or linguistic constructs. Instead of each part of the network learning “a bit of everything,” DeepSeek can isolate specialized behaviors within dedicated experts, preventing different knowledge domains from interfering with one another. Consequently, this approach can yield improvements in tasks that demand deeply specialized reasoning, from complex math problem-solving to niche domain queries. Yet, this structure also adds a layer of complexity: gating and load-balancing algorithms must dynamically route tokens to the optimal experts, all while managing potential imbalances and ensuring stable training (Dai et al., 2024).

#### 1.2.4 Multi-Head Latent Attention (MLA)

To further refine the model’s efficiency, DeepSeek employs Multi-Head Latent Attention, an advanced variant of the multi-head attention mechanism (Shao et al., 2024; Shazeer, 2019; Ainslie et al., 2023). MLA compresses key–value caches by as much as 80–95%, enabling the model to handle long sequences or multiple queries in memory-scarce environments. This compression involves processing hidden representations through latent vectors, effectively capturing the essential information needed for attention while discarding redundant data. The result is an attention mechanism that retains high fidelity to the original context but demands a fraction of the storage costs.

Implementing MLA calls for a carefully orchestrated pipeline: each head not only learns to focus on distinct aspects of the input but also interacts with a shared latent space that helps aggregate or “summarize” relevant information. In practice, this means DeepSeek can maintain a wide range of attention heads without incurring the usual exponential growth in memory. The overall effect is especially beneficial for tasks involving longer text passages or chain-of-thought reasoning, where any memory bottleneck could otherwise degrade performance or inflate hardware requirements.

#### 1.2.5 Optimization and training process

DeepSeek-R1’s training strategy is explicitly designed to nurture robust reasoning capabilities. First, the model undergoes cold-start fine-tuning on a highly curated dataset, incorporating Chain-of-Thought (CoT) annotations from earlier versions (e.g., DeepSeek-V3). This ensures that even the initial state of DeepSeek-R1 has a solid grasp of systematic reasoning patterns before moving to more extensive optimization. Next, the training proceeds through reasoning-oriented reinforcement learning (RL), where newly generated supervised fine-tuning (SFT) data—obtained via rejection sampling—helps refine the model’s outputs (Guo et al., 2025).

After this first round of RL, a second RL phase broadens the scope to tasks like factual QA, writing, and self-cognition. During these stages, Multi-Token Prediction (MTP) further boosts efficiency by predicting multiple tokens simultaneously rather than in a purely sequential manner (Liu et al., 2024; Gloeckle et al., 2024). Notably, the knowledge gained by DeepSeek-R1 can then be distilled into smaller dense models, such as Qwen-2.5B (Qwen, 2024) or Llama (Dubey et al., 2024), often leading to improved performance over undistilled baselines (Guo et al., 2025). This final step highlights a growing trend in large language model research: even if a model reaches extraordinary size (and performance), intelligently transferring its learned representations into more compact architectures can yield widespread practical benefits, especially where computational or memory budgets are constrained.

### 1.3 ChatGPT architecture

#### 1.3.1 Overview

The architecture of ChatGPT is based on the transformer framework (Vaswani et al., 2017; Brown et al., 2020), utilizing self-attention layers to predict upcoming token in long-range dependencies and keeping up with contextual relationships.

Employment of decoder-only transformer architecture enables unidirectional autoregressive text generation (Wu et al., 2023). Inputs require process of token embeddings along with positional encodings (Maktabdar Oghaz et al., 2025), which could then go to stacked layers of transformer decoder blocks. After processing decoder blocks, the outer layer goes through SoftMax, predicting probability of distribution for the next token. ChatGPT generates only one token at a time, and the previous output becomes automatically part of the next input. After an autoregressive generation, followed by multi-stage training for more aligned response of the model.

#### 1.3.2 Transformer decoder layers

ChatGPT is based on the Transformer architecture (Vaswani et al., 2017). The model allowed natural language processing (NLP) (Xiao and Zhu, 2023) to analyze relationships between words in a sequence, by employing self-attention mechanisms (Naik et al., 2024). GPT models use only the decoder portion (Suresh and Shunmugapriya, 2024) for autoregressive tasks such as text generation, while the usual Transformer model consists of encoder-decoder structure. Based on the prior context, this structure allows ChatGPT to predict the next word in a sequence.

Text input from the users is first tokenized using Byte Pair Encoding (BPE) (Rico Sennrich et al., 2016) or as a variant of SentencePiece (Kudo and Richardson, 2018), and positional embedding is incorporated for the order of tokens (Su et al., 2024). The model operates with multiple layers of masked multi-head self-attention, feed-forward neural networks (FFN), layer normalization, and residual connections. Tokens (sub-word units) are processed sequentially, allowing the model to generate coherent and contextually relevant responses.

## 2 Training methodologies and comparative analysis

This section provides an integrated examination of both the training processes and comparative strengths of DeepSeek and ChatGPT. By exploring these dimensions together, we can better understand how their distinct training approaches contribute to their respective capabilities and limitations in real-world applications.

### 2.1 Training approaches and their impact on model capabilities

#### 2.1.1 DeepSeek: reinforcement learning on the base model

DeepSeek-R1 mainly adopts reinforcement learning (RL), using DeepSeek-V3 (Zhu et al., 2024) as a base model and employing Group Relative Policy Optimization (GRPO) for better reasoning.

Reinforcement learning (Ghasemi and Ebrahimi, 2024; Hamid and Braun, 2019) is a powerful algorithmic framework for optimal decision-making in sequential processes. Unlike supervised learning which relies on labeled data, RL is characterized by a goal-directed agent interacting with an environment to maximize cumulative rewards through trial and error learning (Meng et al., 2020; Sutton, 2018; Omiye et al., 2023). The core components of RL include: the agent (the decision-maker), environment (the context in which the agent operates), state (the current situation), action (what the agent can do), reward (feedback on action quality), and policy (the agent’s strategy). In a typical RL setup, the agent engages with the environment through integrated sensors and actuators. The sensors supply the agent with data about the environment’s current state, while the actuators allow it to perform actions that alter this state. This continuous interaction produces feedback that guides the agent toward optimal behavior (Omiye et al., 2023; Hamid and Braun, 2019). This learning-from-experience approach makes RL particularly suitable for complex, uncertain tasks requiring adaptability (Ghasemi and Ebrahimi, 2024; Meng et al., 2020; Sutton, 2018; Omiye et al., 2023), which explains why DeepSeek employs this approach for its reasoning capabilities.

Applying direct RL to the base model (DeepSeek-V3-Base), DeepSeek-R1-zero cultivates complex reasoning behaviors and bypasses conventional steps of supervised fine-tuning methods. A direct approach to a large scale of RL process allows the model to learn reasoning skills through interaction and feedback. This self-evaluation process in solving complex problems allows the model to explore CoT reasoning (Liu et al., 2024).

Unlike DeepSeek-R1-zero, DeepSeek-R1 employs a multi-stage training process rather than pure RL. That is, using cold start fine-tuning with a small amount of high-quality data, providing a better starting point for the model (Guo et al., 2025). Beginning with some initial knowledge from cold-start data focused on reasoning, the process provides room for development on issues with poor readability and language mixing, which was shown in DeepSeek-R1-zero.

Data outputs fine-tuned go through reasoning-oriented RL, which is large scale RL focusing on enhancing outputs in reasoning tasks. Language mixing is seen in this process, but it is necessary for better alignment in outcome of human preferences. RL is applied to fine-tuned model to achieve desirable outcomes.

For reasoning data, rejection sampling operates at the checkpoint on RL process. This expands dataset using generative reward model for judgement on DeepSeek-V3 model (Guo et al., 2025; Liu et al., 2024). Non-reasoning data like writing, factual QA, self-cognition, and translation (Guo et al., 2025), portions of SFT dataset is reused from DeepSeek-V3. The quality of the responses is evaluated through reward sampling, within the duration of two epochs on the dataset. Total of 600 K and 200 K of samples were collected on reasoning-data and non-reasoning data, respectively (Guo et al., 2025).

For better alignment with human preferences, a second RL phase further refines the overall quality of the LLM with large scale RL on all scenarios. On the second RL phase, tasks are widely incorporated beyond the initial focus and go through a reward system for better convergence with human preferences. It then optimizes goals for improvement on the performance in a wide range of domains and then undergoes generalization to ensure the model’s ability on its reasoning skills.

Group Relative Policy Optimization (GRPO) (Guo et al., 2025; Shao et al., 2024), a novel RL algorithm developed by DeepSeek, is applied to save the training cost of the model. Unlike traditional RL methods, critic model, which is almost the same size as a policy model, is not required when GRPO is applied, reducing computational requirements. GRPO employs a group-based approach, in which the model generates multiple responses to a single query and relative responses are grouped and compared. Instead of having absolute rewards from a critic model, the advantage is estimated relatively based on the performance within the group. This interaction within the environment enables the model to optimize its actions pursuing better outcomes. To stabilize the learning process and prevent radical policy changes, a KL-Divergence penalty is incorporated to GRPO.

#### 2.1.2 DeepSeek: reward modeling

DeepSeek adopts a carefully designed, rule-based reward framework to guide its reinforcement learning (RL) process and ensure that generated responses meet specific quality benchmarks (Guo et al., 2025). Unlike purely neural reward models, which risk “reward hacking” where the RL policy exploits loopholes in the learned reward function, a rule-based system provides clearer, more transparent objectives. By encoding explicit criteria for correctness and clarity, DeepSeek aims to promote consistent improvements in key performance areas—particularly in tasks demanding high degrees of factual accuracy and structured reasoning.

Nevertheless, employing such a rules-based approach comes with its own trade-offs. On one hand, it can offer more predictable and stable rewards for well-defined tasks, such as mathematics or coding, where the correctness of an answer can be objectively verified. On the other hand, purely rule-based systems can struggle to capture nuanced aspects of human communication—like stylistic appropriateness or subtle conversational cues—that might be better learned via human annotations. DeepSeek acknowledges these complexities by dividing its reward mechanism into two core components: accuracy rewards and format rewards. Each addresses a distinct dimension of output quality, making the model’s RL process more holistic and adaptable.

#### 2.1.3 Accuracy rewards

Accuracy rewards serve as the backbone of DeepSeek’s rule-based strategy by judging whether a model output is factually correct, especially in deterministic tasks like math problems, coding challenges, or structured data queries (Guo et al., 2025). For instance, math outputs must enclose the final answer in a designated format (e.g., within a box), a requirement that enables a straightforward verification method. The system can thus confirm correctness without relying on subjective human feedback.

Similarly, coding tasks often rely on tangible benchmarks, such as compiling code or matching output to known test cases, to ascertain accuracy. By automating these checks, DeepSeek can rapidly iterate through multiple candidate solutions and reward only those that satisfy clearly established criteria. This approach streamlines the RL loop and reduces the risk of ambiguous or unverifiable outputs—common pitfalls in text-based environments where correctness can be harder to gauge. At scale, these explicit rules help ensure that the model consistently gravitates toward high-fidelity answers, significantly benefiting advanced problem-solving or technical applications where errors can be costly.

#### 2.1.4 Format rewards

While factual correctness is paramount, clarity and structure are also crucial for user-friendly outputs. DeepSeek addresses these needs through format rewards, which encourage well-organized responses and explicit reasoning trails (Guo et al., 2025). The model leverages dedicated tags—such as <think> and </think>—to delineate internal reasoning segments from the final answer. These tags compel the model to present its chain-of-thought more transparently, making it easier for both automated systems and human reviewers to follow the logic behind each conclusion.

Such structured output not only benefits general readability but also supports downstream tools that might parse or score model outputs. For instance, a separate utility can look for <think> sections to assess the reasoning steps, while end-users can simply focus on the final sanitized answer. In essence, format rewards institutionalize best practices in explanation and documentation. By linking these practices to tangible benefits in the RL reward signal, DeepSeek encourages the model to consistently produce outputs that are both accurate and neatly structured—thereby mitigating some of the confusion and opacity that often accompany large language models.

#### 2.1.5 ChatGPT: training strategies and human feedback

In contrast to DeepSeek’s RL-centric approach, ChatGPT employs a multi-stage training process with significant emphasis on human feedback for alignment. Reinforcement Learning from Human Feedback allows ChatGPT to have more conversational features as an LLM. Through three key training phases, refinement of data trains the model’s tendency to have more favorable outcomes.

##### 2.1.5.1 Pre-training phase

Pre-training of the data is to train the model reasoning patterns, grammar, basic knowledge, and priority of tasks without labeling (Hariri, 2023; Naik et al., 2024). Using datasets of diverse corpora, pre-training aims to minimize cross-entropy loss on predicting upcoming token, by learning the pattern statistically (Hariri, 2023). In this process, larger variants require distributed training throughout the GPU.

##### 2.1.5.2 Supervised fine-tuning phase

Supervised Fine-Tuning (SFT) is to train the model to come up with desirable outcomes and tones (OpenAI, 2024). Human reviewers rank the outputs of the model, writing high-quality samples for better alignment. This process allows the model to have conversational flow along with following natural instructions. This stage represents a significant divergence from DeepSeek’s approach, as ChatGPT relies heavily on human-labeled examples to guide its learning process.

##### 2.1.5.3 Reinforcement learning from human feedback

Reinforcement Learning from Human Feedback (RLHF) allows the model’s output to align with human preferences (Christiano et al., 2017). The process follows SFT by having human trainers evaluate model outputs, and a separate neural network is trained to score the quality of responses by comparing outputs ranked by humans (Ouyang et al., 2022; Stiennon et al., 2020). To refine the learning process and review scores, a KL-Divergence penalty is incorporated into Proximal Policy Optimization (PPO) (Schulman et al., 2017), preventing excess deviation from the original supervised model. Unlike DeepSeek’s rule-based reward system, ChatGPT’s reward function is itself a learned model that attempts to capture more subjective aspects of quality, which can lead to more natural-sounding outputs but may be less predictable in specialized technical domains.

### 2.2 Architectural and capability comparison

#### 2.2.1 General architecture

DeepSeek adopts an open-source framework primarily grounded in reinforcement learning, leveraging design elements like Mixture of Experts (MoE) and advanced attention mechanisms to achieve cost-effective performance in tasks that require deep reasoning or structured problem-solving. By focusing on specialized workflows such as mathematical computations, coding, and technical queries, DeepSeek often stands out for its precision, transparency, and relatively low operational overhead. This positions it well in scenarios where budgets are tight or where domain-specific tasks demand closer inspection of the model’s decision-making process.

In contrast, ChatGPT operates as a proprietary system geared toward general-purpose language generation, conversation, and broad coverage across a variety of domains (OpenAI, 2022). Its architecture is more densely parameterized and is shaped by extensive supervised fine-tuning (SFT) plus Reinforcement Learning from Human Feedback (RLHF). This combination yields a fluent, context-aware text generator capable of adapting to diverse user inputs—from writing assistance to customer support chatbots. However, it tends to require significant computational resources, reflecting the trade-off between broad linguistic capabilities and operational costs.

As summarized in Table 1, DeepSeek demonstrates considerable advantages in **cost-**effectiveness at scale, while ChatGPT’s large-scale pre-training and dense parameter approach lends it superior general-purpose versatility. Practitioners aiming to automate specialized tasks—like debugging or structured decision-making pipelines—may find DeepSeek’s open-source environment more appealing, especially when they require insights into the model’s internal operations and cost management. By contrast, large enterprises that deal with wide-ranging user interactions and prioritize polished, intuitive conversational features are likely to favor ChatGPT.

**Table 1:** DeepSeek and ChatGPT comparison.

	DeepSeek	ChatGPT
Data train	Multi-stage learning Including Reinforcement Learning (RL)	- Supervised Fine Tuning (SFT) - Reinforcement Learning from Human Feedback (RLHF) - Uses pre-trained data
Model used	Mixture-of-Experts (MoE) architecture with DeepSeek-V3 model	Transformer architecture (neural network)
Price (API usage)	Input: $0.14 to $0.17 per million tokens Output: $2.19 per million tokens	Input: $7.50 per million tokens Output: $15.00 per million tokens
Price (Development costs)	$5.5 million to $6 million	Over $100 million
Price (Scaling costs 100 M tokens/month)	About $2,000	About $9,000
Limitations	- Less fluent in normal everyday conversations - In complex operations, additional computational power is required - Primarily well-structured for analytical tasks	- Biased outputs or inaccurate information could be generated: hallucination - Showing weakness in extended conversations - Less effective in logical or highly technical tasks
Future direction	Potential of further development through open-source and customization	Improving conversational abilities and general knowledge, upgrading user-friendly aspects
Privacy and Security	- Data storage servers belong to China, raising concerns about data access - Privacy policy allows sharing with third parties and indefinite storage of data	- Collects significant user data but stored in stronger privacy protection country
Ethics	Potential misuse of data possible	More established guidelines in ethics

**Table 2:** Possible application case.

	DeepSeek	ChatGPT
Software development (Zhu et al., 2024; Chen et al., 2021)	- *Coding*: generates optimized, efficient code - *Debugging*: good at detecting and fixing bugs in codebases	- *Coding*: provides high level of explanations of code - *Debugging*: just explains errors on codes while suggesting alternative solutions
Writers	- Cross references facts & citations - Not powerful at content creation, just process complex arguments and detects inconsistencies	- Suggest alternative narratives or reword - Good at generating ideas, improving readability, content structuring
Businesses	*Customer support*: automated ticket solutions and processing backend data *Financial automation & forecasting*: prediction through ML models	*Customer support*: generates human-like responses *Financial automation & forecasting*: Finding actionable business strategies through data
Healthcare (Omiye et al., 2023)	Process medical history and provide preliminary assessments during telemedicine	Interacts with patients in real-time during telemedicine
Education (Mahyoob and Al-Garaady, 2025)	*Grading*: Grades structured answers *Personalized tutoring*: generates custom problems for each students *Summary*: Extracting key points from lectures *Language learning*: provide more succinct explanations	*Grading*: Gives human-like feedback *Personalized tutoring*: answering to conceptual questions *Summary*: explaining things in simple terms *Language learning*: offers elaborate answers
Generative arts	*AI-Generated Visuals*: Detail refinement of the image *Music composition*: make musical pattern *Scriptwriting*: op	*AI-Generated Visuals*: Describes the detail of image *Music composition*: structures lyrics on emotions and themes *Scriptwriting*: op
Gaming	Ensuring consistency in mechanics	NPCs are dynamic and human-like
Cryptocurrency	*Blockchain*: generates transaction analysis	*Blockchain*: teaches crypto concepts

The insights from Tables 1–3 also highlight a strategic dimension: an organization’s choice of LLM depends not only on raw performance metrics but also on how the model’s architecture aligns with operational goals, budget constraints, and domain requirements.

**Table 3:** Benchmark performance.

	DeepSeek-R1 (Guo et al., 2025)	ChatGPT (GPT-4) (Achiam et al., 2023)
MMLU	90.8%	86.4%
GSM8K (Manik, 2025)	~88%	92%
Human Eval.	N/A	67%
Code forces	~2029	~2061
AIME (Math)	79.8%	79.2%

On domains such as coding, especially on Python, DeepSeek outperformed in correctness (Manik, 2025), whereas ChatGPT showed faster response and higher accuracy in scientific computing (Jiang et al., 2025). DeepSeek excels in targeted tasks such as algorithmic coding, whereas ChatGPT leads on broad knowledge benchmarks and speed. DeepSeek’s architecture shows high performance on reasoning benchmarks such as GSM8K and MMLU (Manik, 2025). The fact that no single model dominates in every field is backed up by the fact that in clinical healthcare, the performance of ChatGPT was verbose while DeepSeek showed relative conciseness (Mondillo et al., 2025). In comparison of the two models in language education, DeepSeek performed high accuracy in grammar correction, while ChatGPT exceled in superior reasoning capabilities such as context understanding and overall adaptability in language learning scenarios (Mahyoob and Al-Garaady, 2025). In business writing, ChatGPT was more capable of performing persuasive tone modulation, business communication, and content creation while DeepSeek excelled in grammar precision and factual consistency (AlAfnan, 2025). Teams must therefore strike a balance between a transparent, specialized system (DeepSeek) and a more universally applicable but potentially costlier platform (ChatGPT).

#### 2.2.2 Transparency and explainability

DeepSeek’s open-source ethos inherently provides a higher level of transparency: developers can examine and modify the codebase, while also gaining clearer visibility into how the model arrives at certain conclusions. This is particularly advantageous for specialized sectors, such as healthcare or finance, where traceability of decision-making and adherence to regulatory standards can be critical. However, while DeepSeek offers openness, it does not offer full transparency as training data and code is disclosed to public (Sapkota et al., 2025). “Open-washing” could be an issue in this part, which means although the model is built on open-source, it is not fully transparent (Sapkota et al., 2025). This could lead to underdevelopment and limited utility of the model with lack of full transparency. Nevertheless, DeepSeek’s capacity for “natural-sounding” explanations is more limited, relying instead on structured reasoning tags and rule-based reinforcement that do not always translate into effortless, human-like narratives.

By contrast, ChatGPT excels in producing fluid, conversational explanations that emulate human dialogue. This strength stems from extensive fine-tuning on diverse text corpora, which encourage generative fluency. However, ChatGPT’s closed-source nature means users have no direct access to the underlying architectural details or training data, complicating deep audits of its reasoning. While it can “explain” outputs in a user-friendly manner, these explanations are not necessarily grounded in an open, inspectable mechanism. As a result, organizations needing to audit the chain of logic behind each inference may find ChatGPT insufficiently transparent for certain mission-critical applications.

#### 2.2.3 Ethical considerations

Because DeepSeek is open-source, it leaves much of the ethical responsibility to the user. Developers must ensure that their deployments respect data privacy, regulatory requirements, and content guidelines, as DeepSeek does not come preloaded with the same robust guardrails or usage restrictions often found in large, proprietary models. This openness can be a double-edged sword: it fosters innovation and customization while simultaneously presenting potential risks if proper safeguards are not put in place.

ChatGPT, on the other hand, is packaged with more built-in ethical and safety guidelines stemming from its commercial origin and human-in-the-loop fine-tuning process. Through RLHF, the model learns to curb overtly harmful or biased content, though it remains prone to producing errors or offensive text if not prompted responsibly. It administrates safeguards with harmlessness criterion to reduce toxicity (Achiam et al., 2023). While these additional safety features can enhance user trust, they do not fully eliminate biases or inaccuracies. Moreover, ChatGPT’s reliance on large-scale user data raises its own privacy questions, as the system often logs interactions for continuous improvement. In short, each approach—open-source autonomy vs. proprietary guardrails—comes with its own set of ethical considerations that must be weighed against an organization’s compliance requirements and societal impact goals.

#### 2.2.4 User experience

In terms of direct user interaction, DeepSeek typically prioritizes task-specific accuracy and structured responses. This approach can be ideal for analytical jobs, code generation, or any use case demanding precise, reproducible answers, albeit sometimes at the expense of slower response times or a steeper learning curve for prompt engineering. Users with specialized technical backgrounds may appreciate the fine-grained control over outputs and the ability to tune the system more exactly to their needs.

ChatGPT, in contrast, offers a broadly appealing user experience characterized by quick, coherent dialogue and extensive language coverage. It can handle casual queries just as readily as more in-depth discussions, making it an attractive choice for business customer service, educational aids, or creative writing. This seamless responsiveness, however, can mask certain pitfalls—such as hallucinated facts, lack of deep domain expertise, or limited introspection into how the model generated a specific conclusion. Consequently, while ChatGPT can appear more polished and immediate, serious users must remain vigilant about validating outputs.

Overall, deciding between DeepSeek and ChatGPT for a given application involves carefully weighing the importance of transparency, specialized reasoning, cost constraints, and conversational polish. By examining how these factors align with organizational objectives—whether those revolve around budget, regulatory environment, or breadth of user engagement—decision-makers can better identify which model serves their target use cases most effectively.

## 3 Pros and cons of DeepSeek in comparison to ChatGPT

DeepSeek presents a distinct blend of advantages and disadvantages that stem from its open-source, reinforcement-learning-centric design. While the model exhibits notable strengths in advanced reasoning and cost-effectiveness, it also faces limitations in areas like linguistic versatility and multi-turn interactions. Understanding these trade-offs helps potential users and developers determine if DeepSeek aligns with their specific project requirements and operational constraints.

### 3.1 Pros of DeepSeek

#### 3.1.1 Advanced reasoning

DeepSeek’s architecture and multi-stage reinforcement learning pipeline enable heightened capabilities in tasks requiring complex reasoning. By integrating Chain-of-Thought (CoT) prompts (Liu et al., 2024), self-reflection (Kumar et al., 2024), and verification loops, the model systematically refines its outputs until it arrives at a more robust final answer. This structured approach to problem-solving can be especially beneficial in domains where logical precision is critical, such as mathematics, data science, or code generation which is evident on benchmark datasets like AIME (79.8%, pass@1) and MATH-500 (97.3%, pass@1) (Guo et al., 2025). As a result, DeepSeek often outperforms purely supervised models on tasks that require iterative thinking or multi-step reasoning.

#### 3.1.2 Transparency from open source

One of DeepSeek’s hallmark features is its MIT open-source licensing, allowing practitioners to inspect, modify, and customize the underlying codebase. This transparency extends beyond simply reviewing the source; it provides insights into how the model processes information and arrives at conclusions. Researchers and developers can adapt DeepSeek to specific environments, introducing specialized training data or domain-specific modules without being locked into a proprietary framework. Such openness not only fosters community-driven improvement but also supports auditing and debugging, which can be vital for mission-critical applications that demand verifiable accuracy.

#### 3.1.3 Cost-effectiveness

DeepSeek’s adoption of Mixture of Experts (MoE) and other performance optimizations delivers significant savings in both computation and development costs. By activating only a subset of parameters for each token, the model reduces overhead without substantially compromising performance. This efficiency can translate into lower expenditures for training and inference, making large-scale deployments more accessible to organizations operating on tight budgets. Combined with its open-source nature—eliminating expensive licensing fees—DeepSeek emerges as a compelling choice for enterprises and research labs looking to adopt advanced AI solutions without incurring the financial burden typical of similarly sized language models.

### 3.2 Cons of DeepSeek

#### 3.2.1 General capability

Despite DeepSeek’s strengths in technical and reasoning-oriented tasks, it exhibits limited functionality in multi-turn dialogue, complex role-playing, calling external APIs, and generating structured JSON outputs (Guo et al., 2025). This shortfall reflects a narrower design focus; while DeepSeek excels in iterative reasoning, it may not match the versatility of some general-purpose LLMs when faced with fluid, back-and-forth conversational settings or tasks requiring rich context-switching. Ongoing research and additional training data could help expand these capabilities, but for now, users may find the model less adaptable for interactive or dynamic applications.

#### 3.2.2 Language mixing and bias

DeepSeek’s training pipeline is primarily geared towards English and Chinese, which can cause various drawbacks. For one, reliance on bilingual data sometimes results in inadvertent language mixing during inference, harming clarity when monolingual output is desired (Hendrycks et al., 2020). Additionally, the model’s heavy focus on these two languages may introduce lexical or cultural biases, making it less suitable for truly global deployments. Expanding the training corpus to include more languages and dialects could mitigate these issues, but would likely demand significant computational and data-collection resources.

#### 3.2.3 Prompt engineering sensitivity

DeepSeek often requires precise and carefully structured prompts to unlock its full reasoning potential. Brief or ambiguous inputs may trigger suboptimal responses, forcing users to craft more elaborate instructions or manually format queries. While this attention to detail can encourage better user-model interaction, it also elevates the technical overhead for those unaccustomed to prompt engineering. In commercial or enterprise settings, staff may need special training to ensure they phrase their queries in a manner that yields the best results.

#### 3.2.4 Limited gains in software engineering tasks

Although DeepSeek’s iterative reasoning theoretically applies to many domains, software engineering has remained a challenging area, backed up by SWE benchmark numbers at 49.2% (Guo et al., 2025). Large-scale reinforcement learning has not been extensively tested in code-centric tasks beyond the scope of DeepSeek-V3, meaning the model’s enhancements in such tasks often plateau. Organizations specifically interested in automated code generation, refactoring, or debugging may find better out-of-the-box performance from models that have been explicitly tuned for these purposes. Further research and dedicated datasets could help DeepSeek close this gap.

#### 3.2.5 Regulatory uncertainty

DeepSeek’s open-source structure and data-hosting policies introduce complexities in jurisdictions that maintain stringent data governance rules. Certain countries and industries—including those with sensitive security requirements—may ban or limit DeepSeek due to its perceived risks, such as potential data leakage or unverified usage in surveillance. These regulatory uncertainties might deter large multinational companies from fully adopting the model, particularly where compliance with privacy and export regulations is paramount. Addressing these concerns would require both technical safeguards (e.g., on-premises deployments, data encryption) and clearer policy guidelines to reassure stakeholders.

## 4 Current challenges of AI (DeepSeek and ChatGPT)

Despite the substantial advancements made by DeepSeek, ChatGPT, and other modern large language models, the broader AI field still confronts a number of formidable challenges that can limit the reliability, safety, and real-world applicability of these systems. The significance of acknowledging these hurdles goes beyond mere academic concern: regulators, practitioners, and end users must all grapple with the consequences that arise when AI deployments fall short of expectations or raise ethical red flags. Although the specific manifestations of these challenges can differ between DeepSeek’s open-source, cost-conscious architecture and ChatGPT’s closed-source, user-friendly design, the following issues broadly encapsulate common obstacles in AI development and use.

### 4.1 General challenges in AI

#### 4.1.1 Data quality

High-quality, representative training data form the cornerstone of any successful AI model. In practice, however, data are often marred by inaccuracies, biases, or gaps in coverage—problems that can create downstream errors and reduce model performance. In domains like healthcare or finance, even small data shortcomings can lead to large-scale inaccuracies, disproportionately harming vulnerable populations or producing misleading results. Moreover, curating, cleaning, and labeling massive datasets frequently requires extensive computational, financial, and human resources, complicating efforts to refine and maintain model quality over time.

#### 4.1.2 Transparency and explainability

AI models—especially large, closed-source ones—can appear as “black boxes,” (Pedreschi et al., 2019) offering little insight into how they process information and arrive at their outputs. While techniques like attention visualization and feature attribution have made incremental progress toward more interpretable systems, fully transparent reasoning remains elusive. In high-stakes sectors such as healthcare, law, or finance, the inability to audit a model’s internal logic can create significant barriers to adoption. Clinicians or legal professionals, for instance, may be reluctant to rely on decisions they cannot independently verify or explain to their stakeholders. The tension between preserving proprietary technology (as with ChatGPT) versus providing open, inspectable source code (as with DeepSeek) underscores how transparency can itself become a competitive differentiator.

#### 4.1.3 Privacy concerns

AI systems routinely ingest and process large amounts of personal or sensitive data. Whether it is a user’s browsing history in a recommender system or confidential patient health records in a diagnostic tool, the stakes for privacy violations are high. Even partial data breaches can cause serious repercussions, from identity theft to compromised research. In some jurisdictions, data privacy regulations such as GDPR impose strict requirements for how personal information is collected, processed, and stored. Meeting these standards often entails not only technical solutions—like differential privacy or secure multiparty computation—but also organizational policies and oversight. With models like DeepSeek that may store data on servers located in specific geographic regions, concerns can be magnified by cross-border privacy regulations and varying local standards.

#### 4.1.4 Job displacement

As AI systems mature and become more adept at performing tasks traditionally handled by humans, industries across the spectrum face mounting concerns about automation’s impact on employment. While some see AI as an opportunity to offload mundane, repetitive tasks and free humans for more creative or strategic work, others view large-scale job displacement as an inevitability. Economists debate the net impact AI will have on labor markets, but it is clear that as models like DeepSeek and ChatGPT grow more capable of drafting technical documents, analyzing data, and even making rudimentary managerial decisions, entire categories of jobs could change or vanish altogether. Policymakers and business leaders thus face pressing questions about how to reskill the workforce and distribute the economic benefits AI generates.

In aggregate, these issues emphasize the fact that powerful AI systems bring both immense promise and commensurate risks. Resolving these challenges will require coordinated efforts among researchers, developers, policymakers, and users to ensure that the next wave of AI advances is both innovative and responsibly harnessed.

## 5 Conclusion

The open-source nature of DeepSeek allows customization development. However, on the downside, it could lead to inconsistent performance depending on how the management of the source is done. DeepSeek confronts security and privacy concerns, especially on sensitive user data. Proper management of user data and ensuring reliability to users is an important task that DeepSeek is confronting. DeepSeek also faces limited access to latest chips that could largely affect the performance of the model due to U. S. restrictions. This could lead to limitations providing computational demands when structuring the model. Along with general features, these matters should be handled.

ChatGPT’s dense model architecture shows strength in general tasks, but further developments require better performance in applications. Managing adequate computational costs and response time would be a challenge. Finding ways to reduce its cost and raising efficiency is an important mission. ChatGPT is also expected to work on a phenomenon called “hallucination” (Emsley, 2023; Hamid, 2024), which provides plausible but incorrect answers to users. Refinement in these aspects would excel the quality and reliability of the model in the future.

AI models are foreseeing a better future with their further development in overcoming technical challenges. Enhancing its performance, reliability, and security would significantly evolve the AI landscape.

This comparative review of DeepSeek-R1 and ChatGPT illustrates how different design philosophies—open-source, cost-conscious reinforcement learning on one hand, and proprietary, user-friendly large-scale fine-tuning on the other—shape each model’s strengths, limitations, and ideal usage scenarios. Specifically:

- *Use Cases and Strengths*: DeepSeek-R1’s open-source nature and specialized reinforcement-learning pipeline make it particularly strong in structured, reasoning-heavy tasks (e.g., complex problem-solving, coding optimizations) where transparency and cost control are paramount. ChatGPT’s dense, closed-source approach, refined through massive supervised fine-tuning and RLHF, tends to excel at general-purpose conversation, creative content generation, and rapid prototyping for diverse user-facing applications.
- *Limitations & Considerations*: DeepSeek’s reliance on precise prompts and its weaker support for multiple languages outside English and Chinese might constrain adoption for broad, multilingual use cases. ChatGPT, meanwhile, can suffer “hallucinations” (Emsley, 2023; Hamid, 2024) under specialized or deeply technical queries if not carefully guided. Both models face ongoing challenges around data privacy, ethical guidelines, and the accurate assessment of results.
- *Implications for Researchers & Practitioners*: Organizations seeking budget-friendly, highly customizable solutions could benefit from DeepSeek’s open-source framework—provided they have the capacity to manage updates and handle advanced prompt engineering. Conversely, teams needing fluent, wide-ranging text generation and minimal setup may prefer ChatGPT, though it comes with higher costs and limited code-level transparency.
- *Future Outlook*: As both systems evolve, we anticipate continued improvement in efficiency (e.g., smaller dense models with strong reasoning), multilingual support, and user-friendly interfaces. Researchers should also look for more sophisticated “explainability” features to address regulatory and ethical concerns. Ultimately, these ongoing refinements will further define each model’s niche in domains ranging from healthcare diagnostics and finance to general customer service and educational tools.

At this moment, each model has its prominent strengths and weaknesses, which makes it hard to say one dominates the other. This paper anticipates future models to incorporate the best of the strengths to expand the frontier of effective AI model.

*Key Takeaway*: By highlighting the trade-offs between open-source versus closed-source licensing, specialized reasoning versus broad coverage, and cost-effectiveness versus turnkey ease of use, this paper underscores that no single LLM dominates across all metrics. Instead, the optimal choice depends on aligning a system’s technical architecture with the specific requirements of the target application—whether those are budget, accuracy, language coverage, or developer control.
